# Sensor Fault Detection and Diagnosis Method for AHU Using 1-D CNN and Clustering Analysis

**DOI:** 10.1155/2019/5367217

**Published:** 2019-09-26

**Authors:** Jingjing Liu, Min Zhang, Hai Wang, Wei Zhao, Yan Liu

**Affiliations:** ^1^School of Electro-Mechanical Engineering, Xidian University, Xi'an 710071, China; ^2^School of Aerospace Science and Technology, Xidian University, Xi'an 710071, China

## Abstract

This paper presents a fault detection and diagnosis (FDD) method, which uses one-dimensional convolutional neural network (1-D CNN) and WaveCluster clustering analysis to detect and diagnose sensor faults in the supply air temperature (*T*_sup_) control loop of the air handling unit. In this approach, 1-D CNN is employed to extract man-guided features from raw data, and the extracted features are analyzed by WaveCluster clustering. The suspicious sensor faults are indicated and categorized by denoting clusters. Moreover, the *T*_c_ acquittal procedure is introduced to further improve the accuracy of FDD. In validation, false alarm ratio and missing diagnosis ratio are mainly used to demonstrate the efficiency of the proposed FDD method. Results show that the abrupt sensor faults in *T*_sup_ control loop can be efficiently detected and diagnosed, and the proposed method is equipped with good robustness within the noise range of 6 dBm∼13 dBm.

## 1. Introduction

With people's increasing attention to energy consumption and greenhouse gas emission, heating, ventilation, and air conditioning (HVAC) will have continuous development in the future decades [1]. As one of the most important devices in HVAC, the air handling unit (AHU) is used to supply the cold or heat air for zones of different requirements [2]. A typical AHU consists of multiple subsystems, including numerous sensors, controllers, and actuators. Any hardware failure or controller error, such as temperature sensor fault and the error in fault-tolerant control [3–5], associated with these subsystems might result in the abnormality of AHU and thus affects the whole performance of HVAC system, leading to a negative impact on energy consumption, thermal comfort, and building maintenance cost [6]. The statistical result made in [7] shows that the publications about fault detection and diagnosis (FDD) of AHU occupied 42% of the total publications, being the most common application scenario.

FDD software identifies anomalies in hardware such as sensors, actuators, and controllers. The fault detection stage determines the presence of any fault, while the fault diagnosis stage details the root cause of an issue. Generally, FDD methods can be divided into three categories [1, 8, 9]: the model-based, rule-based, and data-driven methods. Among them, the data-driven method attracts more attention and becomes the most popular FDD method in the field of HVAC [7]. Unlike the other two methods, the data-driven method is based on historical data, which works without the need for theoretical model [9–14]. Moreover, large volume of running data acquired by building automation systems (BAS) can provide an adequate support for this method, which may be further excited by the big data technology.

As a typical data-driven approach, artificial neural network (ANN) has shown great superiority in FDD of AHU. In the early state, the back propagation neural network (BPNN), trained by pattern residuals, was developed [15]. The well-trained BPNN could detect and diagnose eight complete failures. Then, focusing on the detection of the fixed and drifting biases of sensors, a method combining BPNN with the wavelet analysis is introduced [16, 17], and the training process could be promoted by utilizing fractal analysis in the connection of wavelet analysis and BPNN processes [18]. In these abovementioned methods, the classification function of a well-trained BPNN was used to categorize the collected data and identify the running state of AHU.

However, the correlation between the variables in AHU system has not been well considered in [16–18], and false alarm might arise [17]. To solve this problem, the interrelationship of variables in each control loop was investigated, and the essential conservation relations and models in the system were considered to group the variables [8, 19–21]. Wavelet analysis was still used to preprocess the original data to remove disturbing factors, while BPNN played the prediction role in each variable-group.

The applications of BPNN have proved the effectiveness of neural networks in FDD of AHU, and a better anticipation could be achieved if some higher-performance neural networks are taken advantage of. Focusing on detecting and diagnosing the abrupt sensor faults in supply air temperature (*T*_sup_) loop of AHU, this paper presents a novel FDD method by combining one-dimensional convolutional neural network (1-D CNN) and clustering analysis. Herein, the feature extraction capacity of 1-D CNN is fully used to capture the abnormality in measurements, and the WaveCluster clustering is tightly combined with the 1-D CNN to accomplish both the preliminary fault detection and diagnosis. The proposed method is implemented as follows: (1) Preliminary fault detection stage: the features are extracted from raw data by 1-D CNN and clustered using WaveCluster clustering analysis. Then, the existence of suspicious failures is determined according to the existence of denoting cluster. (2) Preliminary fault diagnosis stage: by analyzing the relative position of denoting cluster and reference subspaces, the cause of the suspicious failure is estimated. (3) Confirmation of candidate faults: remove the suspicious failures with low confidence score and generate the final FDD result. By using this method, the abrupt sensor faults in *T*_sup_ loop of AHU system can be detected and diagnosed efficiently and accurately, and the high processing speed of 1-D CNN and WaveCluster clustering analysis makes this method applicable to online FDD.

## 2. Typical Faults and Variables in AHU

**Figure 1:**
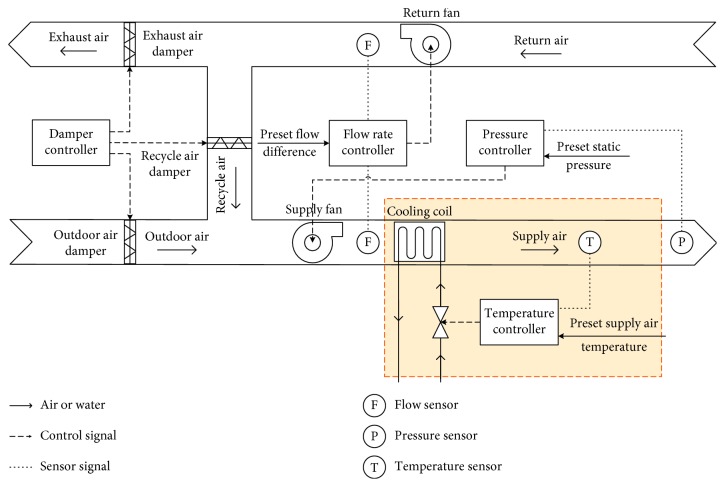
A typical air handling unit.

A typical AHU is shown in Figure 1, where the outdoor air is the input of AHU. First, the outdoor air is provided to AHU and mixed with the recycle air, which is fed back from the multiple zones. Then, after passing through the cooling coil, the mixed air can get a desired temperature. Finally, the air with the desired temperature is supplied to the multiple zones. In the AHU system, the damper controller is used to adjust the flow of air. The supply fan, controlled by a pressure controller, is utilized to modulate the air supply volume. The return fan, controlled by a flow rate controller, regulates the speed of the return air. The temperature of supply air is regulated by a temperature controller.

**Figure 2:**
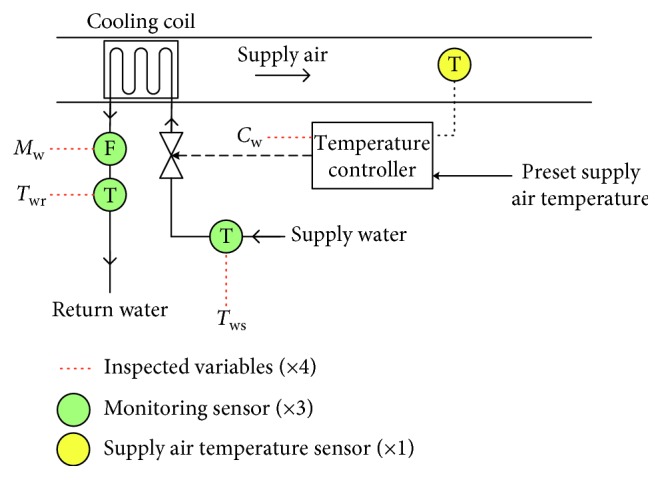
Supply air temperature control loop in AHU.

In this paper, we focus on sensor faults existing in the supply air temperature (*T*_sup_) control loop, which is highlighted with the orange doted box in Figure 1 and detailed in Figure 2. Four typical sensors are considered: supply air temperature (*T*_sup_) sensor, supply water temperature (*T*_ws_) sensor, return water temperature (*T*_wr_) sensor, and chilled water flow rate (*M*_w_) sensor. Output of each sensor or controller is deemed as a variable. In the running state, the variation rule of variables is described as follows.

### 2.1. Normal Case

The *T*_ws_ is assumed unchanged under the normal condition. Through comparing the set temperature of *T*_sup_ with the measured one, the controller outputs a command *C*_w_ to adjust the opening of the water valve, leading to a change of chilled water flow rate *M*_w_ in the coil, and consequently the desired *T*_sup_ can be achieved. In this case, all variables fluctuate smoothly without sudden change.

### 2.2. Faulty Case

Sensor faults investigated in this paper can be divided into two types, the fault of the monitoring variable sensor (*T*_wr_, *T*_ws_, or *M*_w_ sensor) and that of the controlled variable sensor (*T*_sup_ sensor).

#### 2.2.1. Situation 1: Monitoring Variable Sensor Faults

Faults occurred in monitoring variable sensors have no effect on the PID regulation. In this situation, the abnormality only appears on the output measurement of the faulty sensor. Hence, all variables follow the same variation rule as in normal case except the variable of the faulty sensor (*T*_wr_, *T*_ws_, or *M*_w_), which changes abruptly at the failure moment.

#### 2.2.2. Situation 2: Controlled Variable Sensor Faults

Faults occurred in the controlled variable sensor are much more complex. With the feedback control actions, both the *T*_sup_ and its related variables are overlaid with a bias. Take the positive-biased *T*_sup_ sensor fault as an example. It is known that the measured, real, and desired values of *T*_sup_ are the same in normal case. Once the *T*_sup_ sensor fault occurs, the measured value of *T*_sup_ will abruptly exceed its real and desired values. At this point, to maintain the measured value to the desired one, a larger *C*_w_ is generated and thus *M*_w_ increases. Due to the higher refrigeration efficiency led by the larger *M*_w_, the controlled variable *T*_sup_ would gradually decrease until its measured value is equal to the desired one. In this situation, the controlled variable *T*_sup_ changes at the failure moment. Shortly after this moment, the sudden changes of variables *C*_w_, *M*_w_, and *T*_wr_ can be observed almost at the same time.

In conclusion, when no failure is present, no sudden change appears in any of the four variables; when a monitoring variable sensor fails, one of *T*_wr_, *T*_ws_, and *M*_w_ would change abruptly; when the controlled variable sensor fails, variables *C*_w_, *M*_w_, and *T*_wr_ would change abruptly at the same time. Based on this, we select the variables *T*_wr_, *T*_ws_, *C*_w_, and *M*_w_ to detect and diagnose sensor faults in *T*_sup_ control loop and define them as *inspected variables* in this work. Changes of each inspected variable on the occurrence of different sensor faults are summarized in Table 1. Through the analysis of the four inspected variables, the health of the concerned sensors in the *T*_sup_ control loop can be evaluated.

It is worth mentioning that there are two kinds of biases: (1) the measuring bias in the output measurement of the faulty sensor; (2) the bias of variables *T*_wr_, *C*_w_, and *M*_w_ caused by PID regulation when the *T*_sup_ sensor fails. The time of the sensor failure is labeled as *t*_r_, and that of the variable abnormality caused by PID regulation is labeled as *t*_PID_, respectively. To capture the variation taking place at around *t*_r_ or *t*_PID_, defined as *deviance* in this paper, the 1-D CNN-based feature extractor is developed in Section 3.

## 3. Methodology

### 3.1. Outline of the Proposed FDD Method

**Figure 3:**
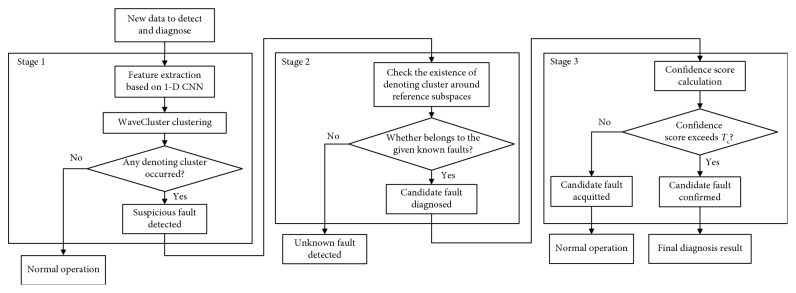
Fault detection and diagnosis logic.

The proposed FDD method using 1-D CNN and WaveCluster clustering is outlined in Figure 3, which contains three stages: preliminary fault detection, preliminary fault diagnosis, and confirmation of candidate faults.

  Stage 1: firstly, the feature extraction based on 1-D CNN is performed to capture sudden changes in variables. Then, through WaveCluster clustering, a preliminary fault detection result can be obtained by judging the existence of denoting clusters, which represents the occurrence of suspicious faults.
  Stage 2: the clustering center of each denoting cluster is calculated. Then, distances between the clustering center and all four reference subspaces are computed and compared with the preset threshold *T*_d_, respectively, each subspace corresponding to a specific sensor fault. The comparison results mark the suspicious sensor fault as a candidate fault or an unknown fault.
  Stage 3: the confidence score of the candidate faults is calculated based on the center of denoting clusters. If the score exceeds the preset threshold *T*_c_, the candidate fault is confirmed as the final diagnosis result. Otherwise, it is regarded that the system is in normal condition.

The proposed FDD method is carried out online, which detects the status of the devices in real time. All data used in this method come from existing sensors and controller of the simulated AHU at running state.

### 3.2. Preliminary Fault Detection Strategy

#### 3.2.1. Feature Extraction Based on 1-D CNN

Convolutional neural network (CNN) [22, 23], which is a class of deep, feed-forward artificial neural network, is widely used in fields like image recognition and object detection for years [24–28]. In recent years, CNN-based fault diagnosis methods are proposed continuously in different fields [29–31]. In these applications, CNN exhibits exclusive feature extraction capacity in finding specific high-level features, based on which the input data can be divided into different categories. For the running data of AHU is a sequence, one-dimensional convolutional neural network (1-D CNN) is used to capture the abnormality in the measurements of variables.

**Figure 4:**
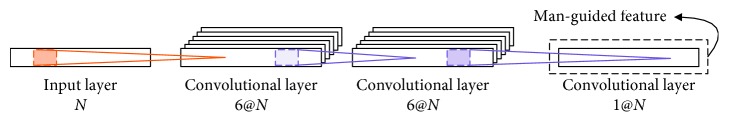
The structure of the 1-D CNN employed in this paper.

The structure of the 1-D CNN employed is shown in Figure 4, which contains three convolutional layers, without pooling layer and fully connected layer. The first layer extracts high-frequency and low-level features. The second and third layers extract higher-level features based on the previous layer. The convolution style of the 1-D CNN is *same*, which means that all feature sequences are in the same length with the input sequence. The proposed 1-D CNN is trained by using a label feature sequence with position information, which characterizes the deviance using a time window, and samples within the window is labeled as “1,” whereas the others are labeled as “0.” During the training process, the output of the last convolutional layer is increasingly closer to the label feature sequence. Instead of automatically exploring features based on the given category information, position guidance is provided for the 1-D CNN in extracting the feature sequence, named *man-guided feature*, which avoids the capture of nondeviance features and reduces the training difficulty.

**Figure 5:**
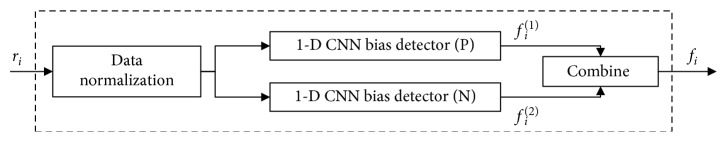
The proposed feature extractor based on 1-D CNN.

The proposed 1-D CNN can handle the bias caused by the faulty sensors and the PID regulation. However, the bias includes positive bias and negative bias, which cannot be processed by a single 1-D CNN. Thus, two 1-D CNNs are used to detect positive bias and negative bias, respectively. Based on the two 1-D CNNs, a feature extractor is constructed, shown in Figure 5, which comprises a data normalization preprocessor, two 1-D CNN bias detectors, and a combiner. The two detectors process normalized data, and two man-guided feature sequences *f*_*i*_^(1)^ and *f*_*i*_^(2)^ are obtained. The combiner is quite simple, which compares the corresponding value of the two sequences one by one and keeps the larger one. Thus, the final feature *f*_*i*_ is achieved.

Benefiting from the simple structure of 1-D CNN, the feature extraction is embedded with fast execution, which contributes to the implementation of high-speed online FDD.

#### 3.2.2. WaveCluster Clustering for Preliminary Fault Detection

WaveCluster clustering is one of the grid-based clustering algorithms, which is often used for multidimensional large-scale data that contain a large number of isolated outliers [32]. Two reasons make WaveCluster clustering especially appropriate for our method: (1) no need to specify the number of clusters, which is suitable for the preliminary fault detection stage where the number of clusters is an important unknown quantity to be observed; (2) high-speed, which meets the requirement of online FDD. In this paper, WaveCluster clustering is used in both preliminary fault detection and preliminary fault diagnosis stages.

**Figure 6:**
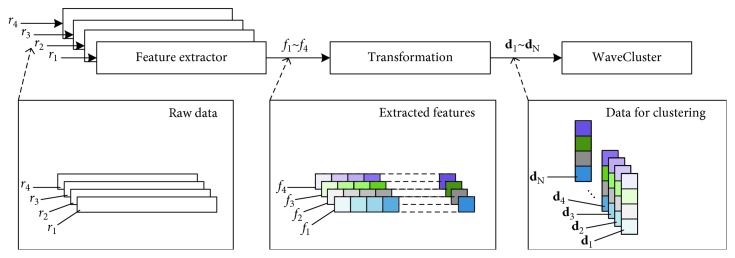
Feature extraction and transformation before WaveCluster clustering.

As the output of the feature extractor cannot be directly used in WaveCluster clustering, a simple transformation is conducted. In this paper, four inspected variables, *T*_wr_, *T*_ws_, *C*_w_, and *M*_w_, are chosen in the *T*_sup_ control loop. Hence, four feature extractors are used. As shown in Figure 6, raw data *r*_1_∼*r*_4_, which are data segments extracted from the four inspected variables, are processed by the feature extractors, respectively. Thus, four extracted features *f*_1_∼*f*_4_ are obtained. The four extracted features are concatenated, yielding the column vectors **d**_**1**_**, d**_**2**_**,…, d**_**N**_, which are fed to WaveCluster clustering.

The column vectors are clustered by WaveCluster, and the number of obtained clusters indicates whether the suspicious fault has occurred. In normal case, only one cluster would occur. We call this cluster *normal cluster*, represented by *C*_*n*_, and *C*_*n*_ usually appears near the origin of the Cartesian coordinate system.

Otherwise, except for the normal cluster, if other clusters also occur, it denotes that one or more suspicious faults are detected. The additionally occurred clusters are named *denoting clusters*, represented by *C*_ *d*_^(1)^, *C*_ *d*_^(2)^,…, *C*_ *d*_^(*k*)^. The number of samples within the denoting cluster is usually very small in comparison with that of the normal cluster. The denoting clusters directly correspond to the suspicious faults, which covers all the sensor faults considered in this paper.

### 3.3. Preliminary Fault Diagnosis Strategy

Fault diagnosis is employed to determine the cause of suspicious faults through analyzing the denoting clusters. This paper considers a total of four sensors, which includes all the key sensors in the *T*_sup_ control loop. The concerned sensors can be divided into two categories, the monitoring variable sensor and the controlled variable sensor. This paper provides separate diagnosis strategies for the two categories. In the diagnosis, a linear space is built by regarding *T*_wr_, *T*_ws_, *C*_w_, and *M*_w_ as the coordinates, and the position of denoting clusters is analyzed in the linear space.

#### 3.3.1. Strategy for Monitoring Variable Sensor

For the monitoring variable sensor faults, the position of denoting clusters is not fixed due to the different level of bias and noise, and it varies within a one-dimension subspace, which we call reference subspace. Subspace of *T*_ws_, *T*_wr_, and *M*_w_ is represented by *R*_*T*_ws__, *R*_*T*_wr__, and *R*_*M*_w__, which are expressed in equations (1)–(3), respectively:

(1) $$\begin{array}{c} R_{T_{\text{wr}}}=\left\{\alpha_{T_{\text{wr}}}={\left(c_{T_{\text{wr}}},0,0,0\right)}^{T}\;|\;c_{T_{\text{wr}}}\in \mathbb{R}\right\}, \end{array}$$

(2) $$\begin{array}{c} R_{T_{\text{ws}}}=\left\{\alpha_{T_{\text{ws}}}={\left(0,c_{T_{\text{ws}}},0,0\right)}^{T}\;|\;c_{T_{\text{ws}}}\in \mathbb{R}\right\}, \end{array}$$

(3) $$\begin{array}{c} R_{M_{\text{w}}}=\left\{\alpha_{M_{\text{w}}}={\left(0,0,0,c_{M_{\text{w}}}\right)}^{T}\;|\;c_{M_{\text{w}}}\in \mathbb{R}\right\}. \end{array}$$

In this paper, we diagnose the monitoring variable sensor faults through checking whether the denoting cluster sits near a certain reference subspace. Taking *C*_d_^(*j*)^ as an example, its clustering center is represented by *μ*_*j*_, which is obtained by averaging all members in the cluster. Then, the Euclidean distance *d*_*V*_^(*j*)^ of *μ*_*j*_ to each reference subspace *R*_*V*_ is calculated from equation (4), where *I* is an identity matrix:

(4) $$\begin{array}{c} d_{V}^{\left(j\right)}={\left\|\left(I-\frac{{\bar{\alpha }}_{V}{\bar{\alpha }}_{V}^{T}}{{\bar{\alpha }}_{V}^{T}{\bar{\alpha }}_{V}}\right)\mu_{j}\right\|}_{2}\text{,}\;\left(V\in \left\{T_{\text{ws}},T_{\text{wr}},M_{\text{w}}\right\},\;{\bar{\alpha }}_{V}\in R_{V}\right). \end{array}$$

If the calculated *d*_*V*_^(*j*)^ is less than or equal to the preset threshold *T*_d_, the denoting cluster *C*_d_^(*j*)^ denotes that the corresponding monitoring variable sensor fails, which is marked as the candidate fault. The three reference subspaces, *R*_*T*_ws__, *R*_*T*_wr__, and *R*_*M*_w__, are orthogonal to each other, and the threshold *T*_d_ is set to a small value, which guarantee the denoting cluster *C*_d_^(*j*)^ has no chance to sit near two or more reference subspaces. However, if none of the three distances is less than or equal to *T*_d_, judgement of the controlled variable sensor fault is needed.

#### 3.3.2. Strategy for Controlled Variable Sensor

When controlled variable sensor fails, it will have great influence on the inspected variables *T*_wr_, *C*_w_, and *M*_w_. In this case, the denoting cluster no longer varies within a one-dimension subspace, but within a three-dimension subspace, which is represented by *R*_*T*_sup__:

(5) $$\begin{array}{c} R_{T_{\sup}}=\left\{\alpha_{T_{\sup}}={\left(c_{T_{\sup}}^{\left(1\right)},0,c_{T_{\sup}}^{\left(2\right)},c_{T_{\sup}}^{\left(3\right)}\right)}^{T}\;|\;c_{T_{\sup}}^{\left(1\right)},c_{T_{\sup}}^{\left(2\right)},c_{T_{\sup}}^{\left(3\right)}\in \mathbb{R}\right\}. \end{array}$$

The dimension of the subspace determines the diagnosis accuracy of the controlled variable sensor. To improve the diagnosis accuracy, the correlation between the four inspected variables is analyzed for the intention of dimension reduction. First, the Pearson correlation coefficient of *T*_wr_, *T*_ws_, *C*_w_, and *M*_w_ is calculated based on equation (6), and the results are shown in Table 2.

(6) $$\begin{array}{c} r_{p}=\frac{n_{p}\sum_{i=1}^{n_{p}}x_{i}y_{i}-\sum_{i=1}^{n_{p}}x_{i}\sum_{i=1}^{n_{p}}y_{i}}{\sqrt{n_{p}\sum_{i=1}^{n_{p}}x_{i}^{2}-{\left(\sum_{i=1}^{n_{p}}x_{i}\right)}^{2}}\sqrt{n_{p}\sum_{i=1}^{n_{p}}y_{i}^{2}-{\left(\sum_{i=1}^{n_{p}}yi\right)}^{2}}}. \end{array}$$

As shown in the table, the correlation coefficient between *C*_w_ and *M*_w_ is approximately equal to 1, which indicates that there is a strong correlation between the two variables. The correlation coefficients between *T*_wr_ and *C*_w_ and that between *T*_wr_ and *M*_w_ are both about 0.6, indicating a medium degree of correlation. As for *T*_ws_, it shows little correlations with the other three variables. The result in the table is consistent with the following facts existing in a running *T*_sup_ control loop: (1) the *T*_sup_ sensor fault results in sudden changes on variables *C*_w_, *M*_w_, and *T*_wr_ at the same time, while it has no effect on *T*_ws_; (2) according to the feedback control actions, *M*_w_ is directly influenced by the variation of *C*_w_, while the influence of *C*_w_ on *T*_wr_ coacts with other factors.

(7) $$\begin{array}{c} R_{T_{\sup}}=\left\{\alpha_{T_{\sup}}={\left(c_{T_{\sup}}^{\left(1\right)},0,c_{T_{\sup}}^{\left(2\right)},k^{*}c_{T_{\sup}}^{\left(2\right)}+b^{*}\right)}^{T}\;|\;c_{T_{\sup}}^{\left(1\right)},c_{T_{\sup}}^{\left(2\right)}\in \mathbb{R}\right\}. \end{array}$$

Based on the correlation analysis, *R*_*T*sup_ can be simplified as equation (7). Here, *C*_w_ and *M*_w_ are considered to have a linear relationship due to the strong correlation between them, and *k*^*∗*^ and *b*^*∗*^ are constants. A linear regression model is established to estimate *k*^*∗*^ and *b*^*∗*^, and the least square method is employed. The estimation result is *k*^*∗*^ = 0.9938, which is about 1; *b*^*∗*^ = 0.0077, which is about 0. Therefore, *R*_*T*sup_ can be reduced to two-dimension subspace as equation (9).

(8) $$\begin{array}{c} f\left(x_{i}\right)=kx_{i}+b, \end{array}$$

(9) $$\begin{array}{c} R_{T_{\sup}}=\left\{\alpha_{T_{\sup}}={\left(c_{T_{\sup}}^{\left(1\right)},0,c_{T_{\sup}}^{\left(2\right)},c_{T_{\sup}}^{\left(2\right)}\right)}^{T}\;|\;c_{T_{\sup}}^{\left(1\right)},c_{T_{\sup}}^{\left(2\right)}\in \mathbb{R}\right\}. \end{array}$$

Similar to the diagnosis of monitoring variable sensors, the Euclidean distance of *μ*_*j*_ to the reference subspace *R*_*T*_sup__ is calculated, represented by *d*_*T*_sup__^(*j*)^. The calculation of *d*_*T*_sup__^(*j*)^ depends on the projection matrix **P**. Taking two linearly independent vectors **α**_*T*_sup__^(1)^ and **α**_*T*_sup__^(2)^, where **α**_*T*_sup__^(1)^, **α**_*T*_sup__^(2)^ ∈ *R*_*T*_sup__, to construct a matrix **A** = (**α**_*T*_sup__^(1)^, **α**_*T*_sup__^(2)^), the projection matrix **P** of the reference subspace *R*_*T*_sup__ can be calculated by equation (10). Hence, the Euclidean distance *d*_*T*_sup__^(*j*)^ is obtained from equation (11). Then, the calculated *d*_*T*_sup__^(*j*)^ is compared with the preset threshold *T*_d_ to diagnose if *T*_sup_ sensor fault occurs. If not, an unknown failure is recorded. Based on the above strategies, the entire preliminary fault diagnosis procedure is completed.

(10) $$\begin{array}{c} P=A{\left(A^{T}A\right)}^{-1}A^{T}, \end{array}$$

(11) $$\begin{array}{c} d_{T_{\sup}}^{\left(j\right)}={\left\|\left(I-P\right)\mu_{j}\right\|}_{2}. \end{array}$$

### 3.4. Confirmation of Candidate Faults

To verify the reliability of the fault detection and diagnosis strategies, false alarm and missing diagnosis are evaluated. The false alarm refers to the situation that the normal sensor is wrongly detected as faulty sensor, and the missing diagnosis refers that the real fault of the faulty sensor is not successfully diagnosed. Retaining all the candidate faults lowers the occurrence of missing diagnosis, while that of false alarm is relatively frequent. To improve the deficiency, a procedure named *T*_c_*acquittal* is introduced, which aims at lowering the false alarm and searching for the best trade-off between the false alarm and missing diagnosis. *T*_c_ acquittal can cancel some candidate faults by introducing a confidence score to evaluate the candidate faults.

Mark the center of the denoting cluster *C*_ *d*_^(*j*)^ as *μ*_*j*_ = (*μ*_*j*1_, *μ*_*j*2_,*μ*_*j*3_,*μ*_*j*4_)^T^. If a monitoring variable sensor is diagnosed as the cause of a candidate fault, the position of *C*_ *d*_^(*j*)^ in space mainly depends on the inspected variable of this sensor. Hence, the confidence score of the monitoring sensor faults is defined as follows:

(12) $$\begin{array}{c} \left\{\begin{array}{c} s_{T_{\text{wr}}}^{\left(j\right)}=\mu_{j1},\\ s_{T_{\text{ws}}}^{\left(j\right)}=\mu_{j2},\\ s_{M_{\text{w}}}^{\left(j\right)}=\mu_{j4}. \end{array}\right. \end{array}$$

If the *T*_sup_ sensor is diagnosed as the cause of a candidate fault, the position of *C*_ *d*_^(*j*)^ in space depends on three inspected variables: *T*_wr_, *C*_w_, and *M*_w_. At this time, the confidence score of controlled variable sensor faults is defined as equation (13), where *ξ*_*i*_ is the weight assigned to each inspected variable. In this paper, we set *ξ*_1_ = 0.5 and *ξ*_3_ = *ξ*_4_ = 0.25 according to the result of correlation analysis between the variables.

(13) $$\begin{array}{c} s_{T_{\sup}}^{\left(j\right)}=\overset{4}{\underset{\begin{array}{c} i=1\\ i\neq 2 \end{array}}{\sum }}\xi_{i}\mu_{ji}. \end{array}$$

For each candidate fault, its confidence score is compared with the preset threshold *T*_c_. If the confidence score is smaller than *T*_c_, the candidate fault is acquitted. Otherwise, the candidate fault is confirmed.

It is worth noticing that *T*_c_ acquittal will lead to a reduction in false alarm, but correspondingly the missing diagnosis might arise. Therefore, we should adjust the value of *T*_c_ to achieve a best trade-off between false alarm and missing diagnosis.

## 4. Validation

### 4.1. Data Description and Preparation

The simulation is carried out based on TRNSYS, a graphical-based software environment used to simulate the behavior of transient systems, which is used to build and simulate the *T*_sup_ control loop in AHU. A fault generator and a noise generator are added in the simulation. In this paper, the desired *T*_sup_ is set as 15°C. The *T*_ws_ is set as 8°C. The *C*_w_ is a floating number, varying between 0 and 1. In the simulation, the AHU is installed in the city of Ouagadougou in Africa. The meteorological data used are the typical meteorological year (TMY) data of the city, where 8760 hours' data are contained. This paper only considers the FDD of sensor faults in AHU at cooling conditions, so only the data from May to July are used.

To get the training data and test data, the following operations are performed. For data in faulty condition, firstly, from the simulation starting time *t*_ST_, the integrated fault generator generates faults at time *t*_ST_ + Δ*t*, *t*_ST_ + 2Δ*t*,…, *t*_ST_ + *i*Δ*t*,… respectively, until the simulation terminal time *t*_TT_. Here, Δ*t* is set as five hours. Each fault is generated independently, no overlap or interference with each other. All raw data will be generated after simulation, comprising measurements of four inspected variables from *t*_ST_ to *t*_TT_. Then, the data preprocessing is carried out. The raw data are processed to obtain data segments with the length of *N*, and *N* is set as 160 in this paper. Each data segment is obtained by partitioning the raw data that contains the deviance. For data in normal condition, we inactivate the fault generator and partition the simulated raw data in a same way to get the data segments with the same length. Afterwards, the training data of 1-D CNN are selected from the data in May, while the test data are selected from that in June and July. During simulation, the data sampling interval is set as 28.8 seconds.

The performance of the proposed method is evaluated based on the computation of the following four indices:

  False alarm ratio (FAR): the ratio between the number of wrongly detected normal samples and the number of total normal samples
  Missing alarm ratio (MAR): the ratio between the number of undetected faulty samples and the number of total faulty samples
  False diagnosis ratio (FDR): the ratio between the number of wrongly diagnosed samples and the number of total faulty samples
  Missing diagnosis ratio (MDR): the ratio between the number of unsuccessfully diagnosed samples and the number of total faulty samples

### 4.2. Validation of Preliminary FDD

#### 4.2.1. Preliminary Fault Detection

**Figure 7:**
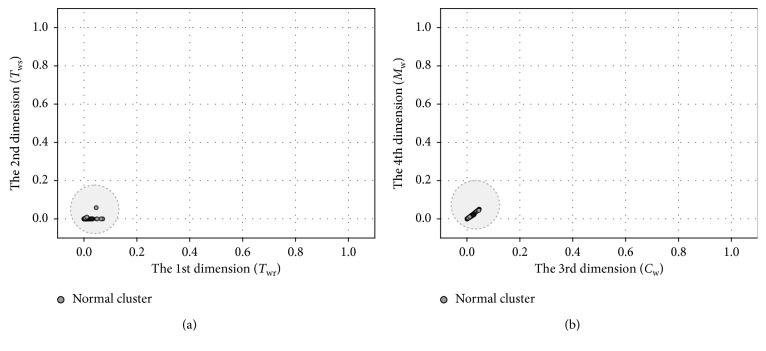
Clustering result in normal condition: (a) 1-2 dimensions; (b) 3-4 dimensions.

**Figure 8:**
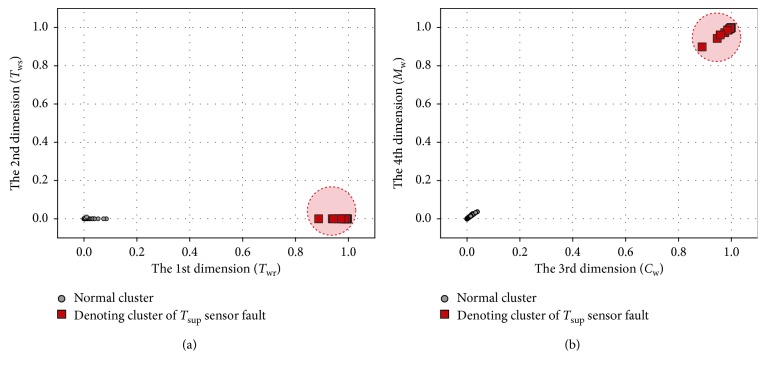
Clustering result in faulty condition: (a) 1-2 dimensions; (b) 3-4 dimensions.

We take the *T*_sup_ sensor fault as an example to show the result of the preliminary fault detection. Here, 0.5°C bias and 6 dBm noise are introduced. Two data segments are detected, which are collected in normal condition and faulty condition, respectively. The results of WaveCluster clustering are shown in Figures 7 and 8, where Figure 7 shows the clustering result in normal condition and Figure 8 shows that of the faulty condition.

As is shown in figures, there is only one cluster generated after the clustering analysis in normal condition, and the position of the cluster is near the origin. In faulty condition, denoting cluster appears which denotes the occurrence of suspicious failures. Besides the *T*_sup_ sensor fault, the other three sensor faults are also simulated. Validation results indicate that denoting cluster also appears under the *T*_wr_, *T*_ws_, and *M*_w_ sensor faults.

#### 4.2.2. Preliminary Fault Diagnosis

**Figure 9:**
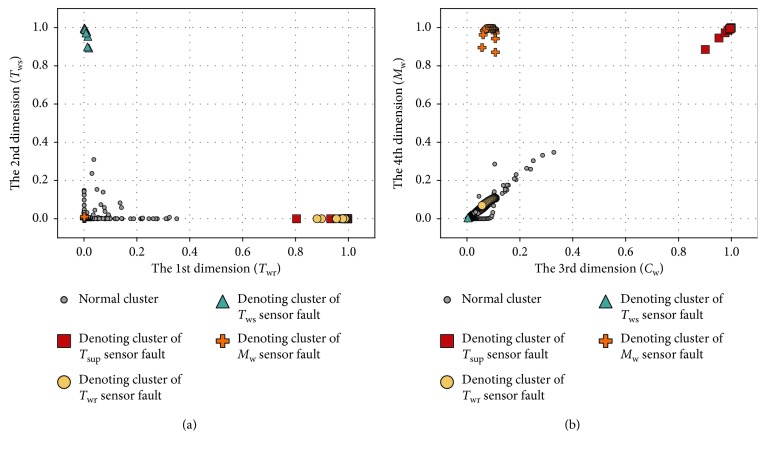
Preliminary fault detection results of all four sensor faults: (a) 1-2 dimensions; (b) 3-4 dimensions.

**Figure 10:**
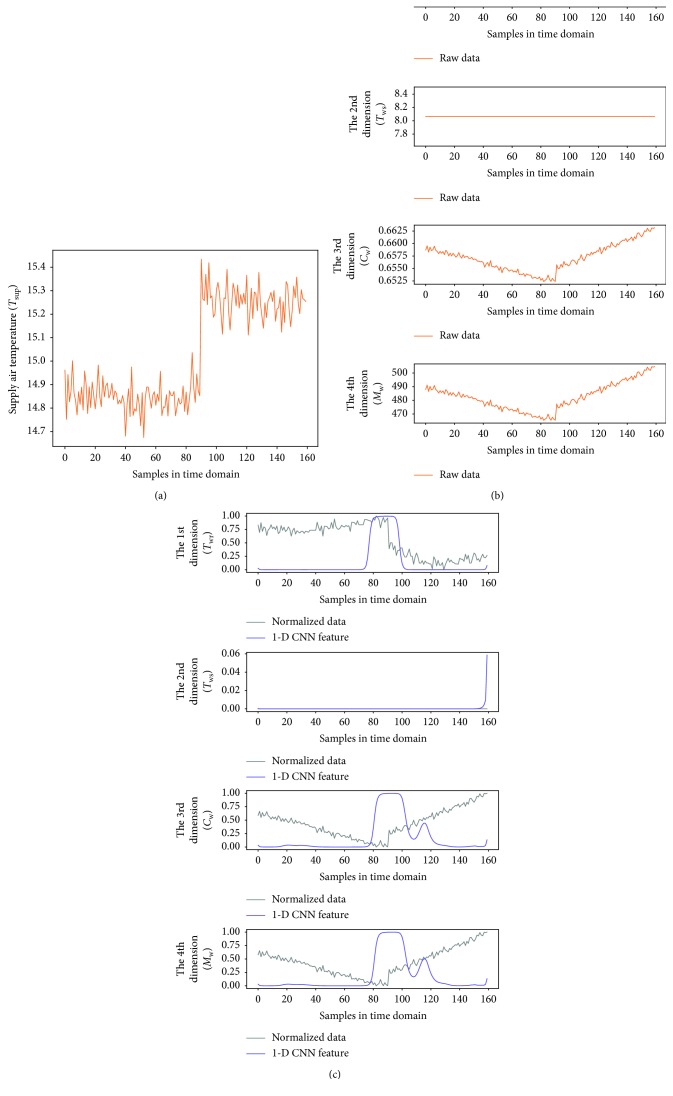
The dynamic of variables in the time domain when *T*_sup_ sensor fault occurs; (a) raw data of the supply air temperature; (b) raw data of four inspected variables; (c) normalized data of four inspected variables and their corresponding 1-D CNN features.

**Figure 11:**
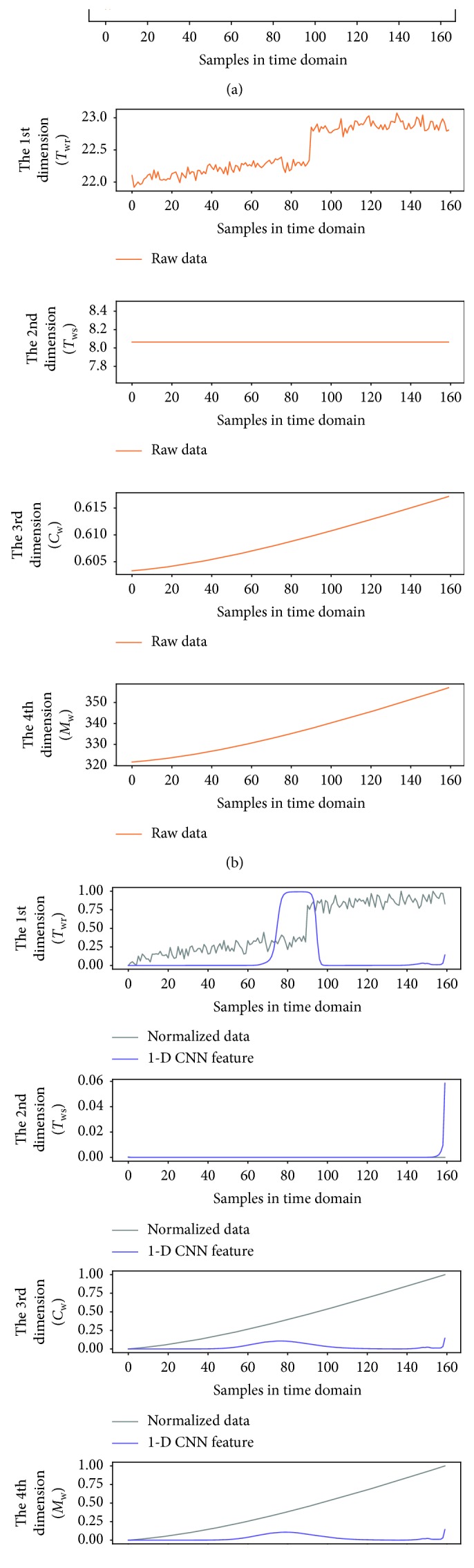
The dynamic of variables in the time domain when *T*_wr_ sensor fault occurs; (a) raw data of the supply air temperature; (b) raw data of four inspected variables; (c) normalized data of four inspected variables and their corresponding 1-D CNN features.

**Figure 12:**
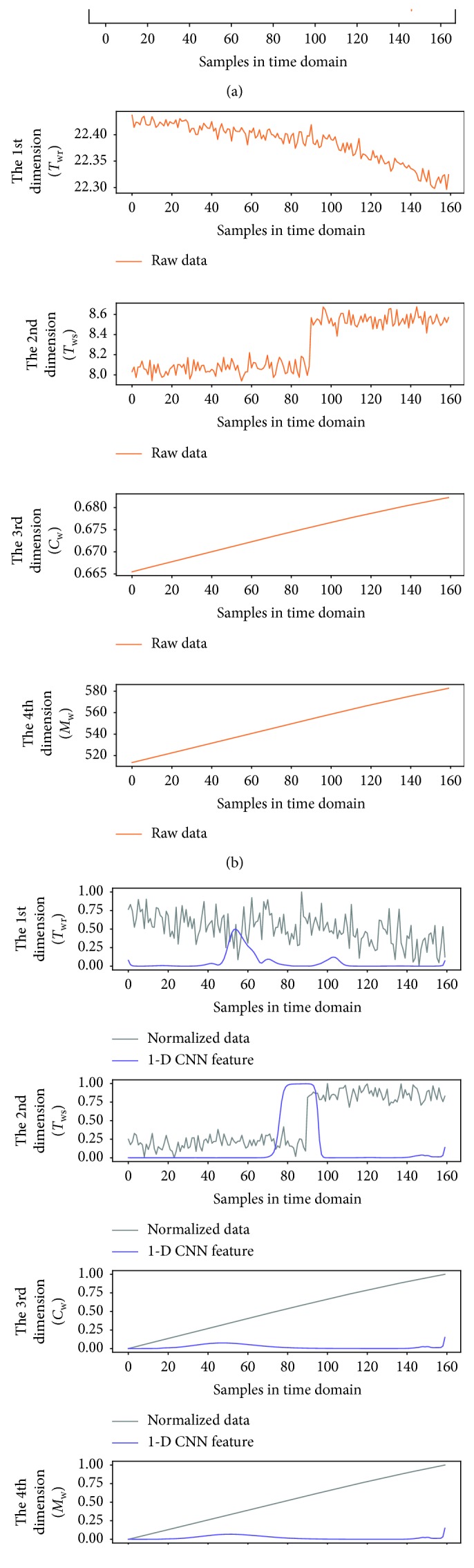
The dynamic of variables in the time domain when *T*_ws_ sensor fault occurs; (a) raw data of the supply air temperature; (b) raw data of four inspected variables; (c) normalized data of four inspected variables and their corresponding 1-D CNN features.

**Figure 13:**
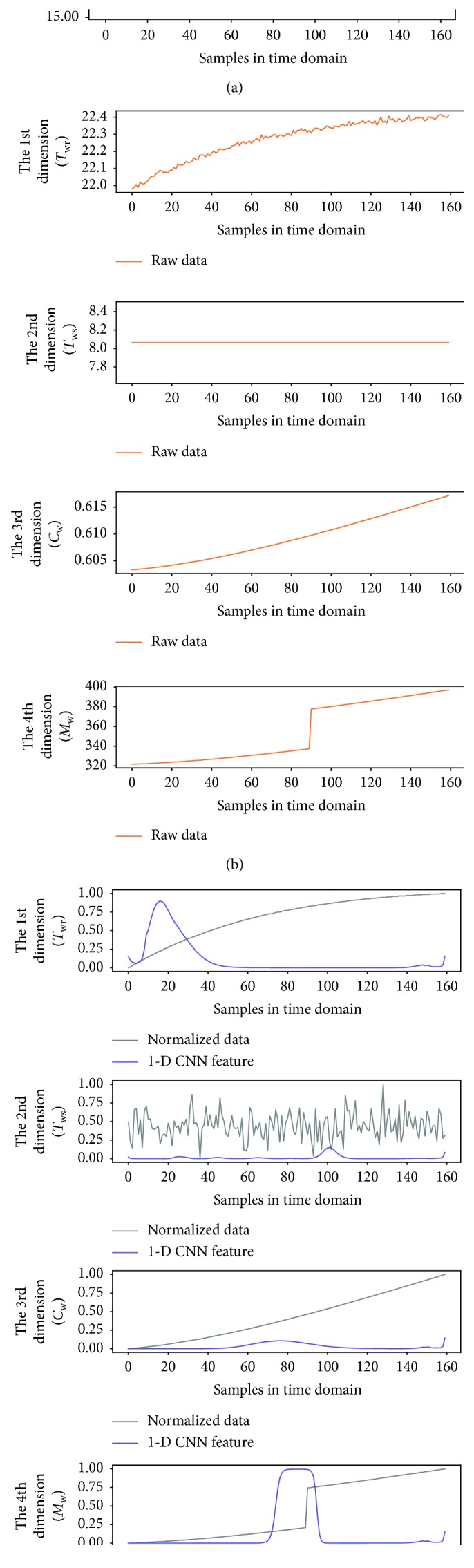
The dynamic of variables in the time domain when *M*_w_ sensor fault occurs; (a) raw data of the supply air temperature; (b) raw data of four inspected variables; (c) normalized data of four inspected variables and their corresponding 1-D CNN features.

To better observe the denoting clusters of different sensor faults, the preliminary fault detection results of all the sensor faults are put in the same space.  Figure 9 illustrates the distribution of the four sensor faults, which is obtained under the 0.5°C biased temperature sensors and 5% biased flow rate sensor, respectively. In addition, a 6 dBm white Gaussian noise (WGN) is added to the output of each faulty sensor during the simulation. We also give the dynamic of variables in the time domain when one of the four sensor faults occurs, as shown in Figures 10–13. In each of these figures, (c) shows not only the normalized data of four inspected variables but also their corresponding 1-D CNN features, which can be correlated with the clustering results shown in Figure 9.

Figure 9 indicates that denoting clusters of different sensor faults are distributed in separate positions. Denoting clusters of the monitoring variable sensor faults sit near the corresponding one-dimension reference subspaces, *R*_*T*_ws__, *R*_*T*_wr__, and *R*_*M*_w__, respectively, while the denoting cluster of the controlled variable sensor fault sits near the two-dimension reference subspace *R*_*T*_sup__. Hence, the threshold *T*_d_ is used to separate the sensor faults.

**Figure 14:**
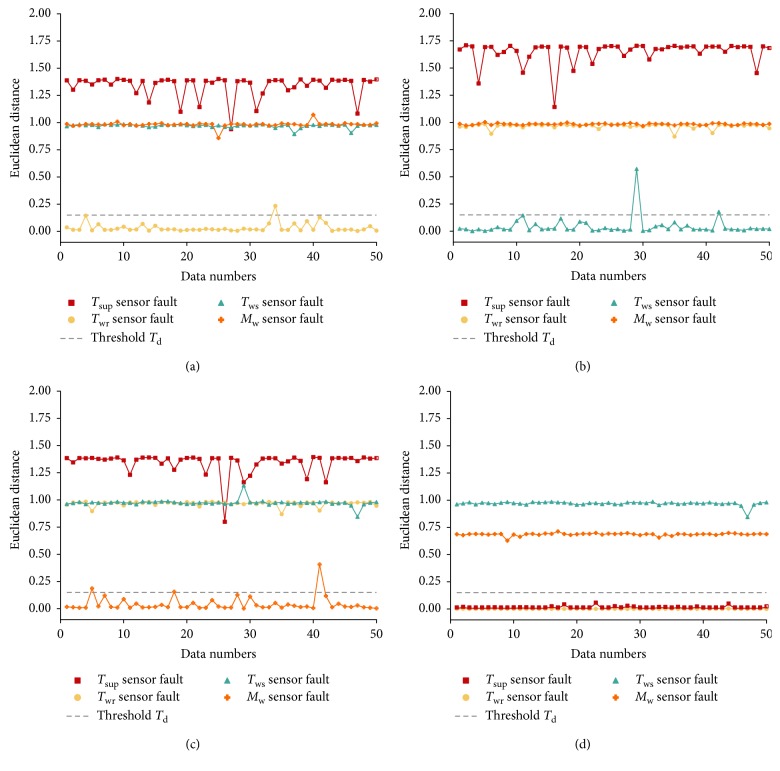
Euclidean distance between four kinds of data and each reference subspace: (a) *R*_*T*_wr__; (b) *R*_*T*_ws__; (c) *R*_*M*_w__; (d) *R*_*T*_sup__.

Experiments are conducted to choose an appropriate value of *T*_d_. In the experiments, data with noise of 13 dBm and biases of 0.5°C and 5% are employed. 50 data are selected for each sensor fault. The distance between the center of each denoting cluster and subspace is calculated and demonstrated in Figure 14, respectively. The figure proves that the denoting clusters of each sensor fault sit very close to their corresponding subspace. One other thing to note is that the denoting clusters of *T*_wr_ sensor faults also sit near the *R*_*T*_sup__ subspace. Since the diagnosis of monitoring variable sensors is carried out before that of the controlled variable sensor, erroneous diagnosis of the *T*_wr_ sensor faults is not introduced. According to the experimental results, we set the threshold *T*_d_ as 0.15, which can accurately diagnose vast majority of the sensor faults.

### 4.3. Searching for the Best Trade-Off between FAR and MDR

Due to variations of noise and bias, the position of the denoting cluster is uncertain. Denoting clusters under different sensor faults are distributed within the corresponding reference subspace of the faults. The proposed method compares the distance between denoting cluster and the reference subspaces rather than the distance between the current-occurred denoting cluster and a certain coordinate. Then the candidate faults are obtained after the comparison.

**Figure 15:**
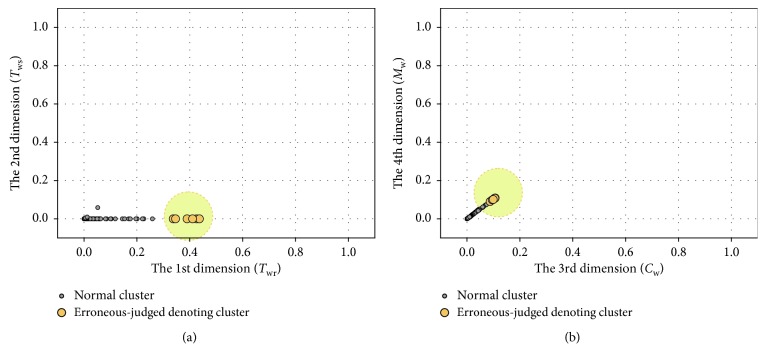
Erroneous judgement case in normal condition: (a) 1-2 dimensions; (b) 3-4 dimensions.

However, sometimes the candidate fault is an erroneous judgement. As shown in Figure 15, the tested data segment is collected in normal condition, but the denoting cluster still appears. Consequently, the fault diagnosis procedure mistakenly announces the occurrence of a *T*_wr_ sensor fault.

Compared with correctly detected denoting clusters, erroneous-judged denoting clusters usually sit closer to the origin. In this case, the confidence score of the candidate fault is relatively low. Hence, the FAR could be decreased through acquitting the faults with low confidence scores. However, that may increase the MDR. The value of *T*_c_ has a significant effect on FAR and MDR, and the response of FAR and MDR to the variations of *T*_c_ is opposite. Hence, experiments are performed to obtain the optimal *T*_c_ that makes the best trade-off between FAR and MDR.

**Figure 16:**
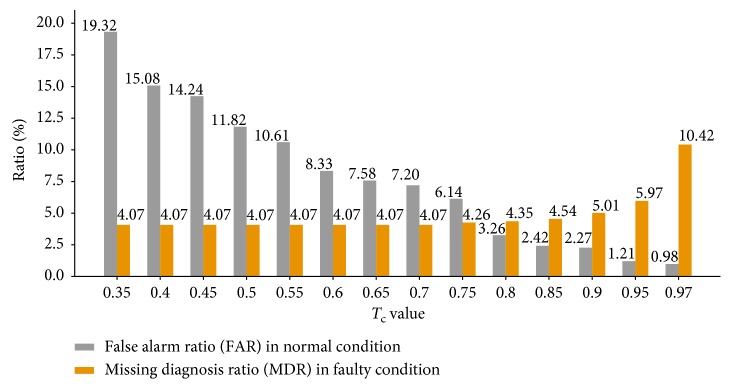
FAR and MDR at different *T*_c_ values.

Using the same batch of data, the proposed method is applied with different *T*_c_, and the FAR and MDR are compared. This batch of data is selected from May, shown in Table 3. As a result, FAR and MDR at different values of *T*_c_ are shown in Figure 16. The MDR remains almost unchanged and slightly increases when *T*_c_ is larger than 0.7. However, the FAR decreases rapidly with the increase of *T*_c_. The figure illustrates that relatively lower FAR and MDR is achieved at the same time when *T*_c_ is 0.85. This *T*_c_ is named the *optimal T*_c_. The optimal *T*_c_ selected in this paper is only an estimate value, which relates to both the amount and the type of the used data.

### 4.4. Performance Evaluation

#### 4.4.1. Detection Efficiency

To validate the reliability of the fault detection strategy, a comparison with existing AHU sensor fault detection strategies based on neural networks is made [8, 21], where indices FAR and MAR are used. The data of our method are obtained through the test data, which includes 1464 hours' raw data, and nearly 14400 data segments are used. In particular, the detection efficiency of different approaches, i.e., the basic neural network (BSC-NN), the auxiliary neural network (AUX-NN), and the combined neural networks (CB-NN), is shown in Table 4, which indicates that the proposed method performs well in both FAR and MAR. Compared with other methods, the proposed method achieves the best detection efficiency in average, which is 0.06% lower than that of the CB-NN proposed in [8, 21]. Especially, the MAR of both of the positive and negative biases of *T*_sup_ sensor faults is 0.00%, which proves that the proposed method has better performance on the controlled variable sensor fault detection.

#### 4.4.2. Diagnosis Efficiency

The diagnosis efficiency of the proposed FDD method is also analyzed, including FDR and MDR. Here, FDR includes two possibilities: (1) the real fault is successfully diagnosed, while one or more inexistent faults are also believed to have occurred; (2) the real fault is misdiagnosed as some other faults. In addition, the performance of preliminary FDD and the FDD with *T*_c_ acquittal is compared, where the optimal *T*_c_ is used. The data used for diagnosis efficiency analysis are the same data used in Section 4.4.1.

Table 5 shows the efficiency, which indicates that, benefiting from the *T*_c_ acquittal, the FDR is significantly reduced. Meanwhile, MDR remains the same in most cases, except for the cases of the *T*_sup_, *T*_wr_, and *T*_ws_ sensor faults with a bias of 0.5°C. The results validate the efficiency of *T*_c_ acquittal.

Table 5 also implies that monitoring variable sensor faults have higher MDR than controlled variable sensor faults. The main reason is that the three inspected variables share the diagnosis of controlled variable sensor faults, which provides more reliable diagnosis results, and only one inspected variable undertake that of monitoring variable sensor faults.

For data with different sensor faults and different biases, the FDD time is approximately the same, being 0.013 to 0.016 seconds. We also test time that it takes to accomplish each one of the stages presented in Figure 3, which is shown in Table 6.

#### 4.4.3. Robustness Test at White Gaussian Noise Condition

This section tests the robustness of the proposed method. The test data used above with biases of 0.5°C and 5% are also used here. FAR and MDR under different noise conditions are shown in Table 7. The test results show that the FAR and MDR have slight variation within a noise range of 6 dBm∼13 dBm, which indicates that the proposed method is equipped with a good robustness under different noise conditions.

## 5. Conclusion

An FDD method for sensor faults in the *T*_sup_ control loop of AHU has been proposed in this paper, in which 1-D CNN and WaveCluster clustering analysis are applied. Four sensor faults are detected and diagnosed, including three monitoring variable sensors and one controlled variable sensor. Validation results show that the proposed method can successfully detect and diagnose the abrupt sensor faults in the *T*_sup_ control loop of AHU. The procedure of *T*_c_ acquittal effectively lowers the FAR and FDR with the cost of a slight increase in MDR, and a best trade-off between FAR and MDR can be obtained at *T*_c_ = 0.85. Robustness assessment indicates that, within a noise range of 6 dBm∼13 dBm, the level of noise shows little influence on MDR and FAR in all conditions.

The limitation of this system is that the adopted WaveCluster clustering algorithm has several hyperparameters, such as grid density and how many outliers can be considered as noise. The set of hyperparameters strongly affects the clustering result. Poor hyperparameters may lead to the absence of real denoting cluster or the presence of erroneous-judged denoting clusters, and eventually affects the final FDD result.

## Figures and Tables

**Table 1:** Effects on each inspected variable when a certain type of sensor fault occurs.

Inspected variables	*T*_wr_	*T*_ws_	*C*_w_	*M*_w_
Sensor fault				
Positive bias in *T*_sup_ sensor	N	—	P	P
Negative bias in *T*_sup_ sensor	P	—	N	N
Positive bias in *T*_wr_ sensor	P	—	—	—
Negative bias in *T*_wr_ sensor	N	—	—	—
Positive bias in *T*_ws_ sensor	—	P	—	—
Negative bias in *T*_ws_ sensor	—	N	—	—
Positive bias in *M*_w_ sensor	—	—	—	P
Negative bias in *M*_w_ sensor	—	—	—	N

Note: “P” means a positive bias of the inspected variable; “N” means a negative bias of the inspected variable; “—” means that the inspected variable is not influenced.

**Table 2:** Result of correlation analysis between the four inspected variables.

	*T*_wr_	*T*_ws_	*C*_w_	*M*_w_
*T*_wr_	1.0000	—	—	—
*T*_ws_	0.1710	1.0000	—	—
*C*_w_	0.5490	0.0722	1.0000	—
*M*_w_	0.5970	0.0634	**0.9880**	1.0000

**Table 3:** The description of the batch of data used to search for the optimal *T*_c_.

Type	Bias	Noise (dBm)	Size of dataset
*T*_sup_ sensor fault	0.5/1.0/1.5	6∼13	264
*T*_wr_ sensor fault	0.5/1.0/1.5	6∼13	264
*T*_ws_ sensor fault	0.5/1.0/1.5	6∼13	264
*M*_w_ sensor fault	5%	6∼13	264
Normal	—	6∼13	1056

Note: in faulty condition, WGNs are added to the output of the faulty sensor during the simulation. In normal condition, WGNs are added to the outputs of *T*_ws_ sensor, *T*_wr_ sensor, and *M*_w_ sensor during the simulation, respectively.

**Table 4:** Detection efficiency of the proposed method and other three existing neural network-based methods.

Type	Bias	Index	BSC-NN (%)	AUX-NN (%)	CB-NN (%)	Proposed (%)
Fixed bias of *T*_wr_ sensor	1.0	MAR	**0.00**	**0.00**	**0.00**	**0.00**
Fixed bias of *T*_ws_ sensor	0.5	MAR	**0.00**	**0.00**	**0.00**	0.25
Fixed bias of *M*_w_ sensor	−5%	MAR	34.60	**0.00**	**0.00**	1.98
Positive fixed bias of *T*_sup_ sensor	1.5	MAR	10.20	4.20	2.50	**0.00**
Negative fixed bias of *T*_sup_ sensor	−1.5	MAR	50.80	**0.00**	**0.00**	**0.00**
Normal	—	FAR	**0.00**	2.44	3.25	3.16
Average	—	—	15.93	1.11	0.96	**0.90**

**Table 5:** Diagnosis efficiency of the preliminary FDD and the FDD with *T*_c_ acquittal.

Type	Bias	The preliminary FDD			The FDD with *T*_c_ acquittal		
		FDR (%)	MDR (%)	Time (s)	FDR (%)	MDR (%)	Time (s)
Fixed bias of *T*_sup_ sensor	0.5	25.28	0.12	0.0154	1.80	**1.67**	0.0156
	1.0	2.77	0.00	0.0153	0.00	0.00	0.0155
	1.5	1.30	0.00	0.0152	0.00	0.00	0.0154

Fixed bias of *T*_wr_ sensor	0.5	25.39	2.59	0.0142	0.17	**2.98**	0.0144
	1.0	25.26	2.47	0.0146	0.22	2.47	0.0149
	1.5	25.26	2.51	0.0150	0.30	2.51	0.0153

Fixed bias of *T*_ws_ sensor	0.5	31.83	4.71	0.0133	7.96	**4.80**	0.0136
	1.0	31.27	5.36	0.0133	8.74	5.36	0.0136
	1.5	30.45	4.41	0.0145	7.35	4.41	0.0147

Fixed bias of *M*_w_ sensor	5%	22.19	6.96	0.0143	12.24	6.96	0.0146

Note: cases that MDR increased are marked in bold.

**Table 6:** Time to accomplish each one of the stages of the proposed method.

Stage	*T*_sup_ sensor fault	*T*_wr_ sensor fault	*T*_ws_ sensor fault	*M*_w_ sensor fault
Stage 1	0.0119	0.0134	0.0138	0.0150
Stage 2	0.0017	0.0021	0.0021	0.0018
Stage 3	0.0002	0.0003	0.0002	0.0002
Total	0.0138	0.0158	0.0161	0.0170

**Table 7:** Efficiency of the proposed FDD method under different noise conditions.

Noise (dBm)	*T*_sup_ sensor fault	*T*_wr_ sensor fault	*T*_ws_ sensor fault	*M*_w_ sensor fault	Normal
	MDR (%)	MDR (%)	MDR (%)	MDR (%)	FAR (%)
6	1.26	2.77	4.84	6.23	4.84
7	1.60	2.77	3.81	4.84	3.11
8	1.48	3.11	3.81	8.30	2.77
9	1.84	3.11	4.50	6.23	4.84
10	1.47	3.11	3.46	7.96	1.38
11	1.47	2.42	6.23	7.96	2.42
12	2.06	3.46	5.54	7.61	2.42
13	2.06	3.11	6.23	6.57	3.46

## Data Availability

The data used to support the findings of this study are available from Jingjing Liu (liujj@stu.xidian.edu.cn) upon request.
